# Difficult to manage the concerns of parents of a scizophrenic patient related to side effects of clozapine

**DOI:** 10.4103/0019-5545.43626

**Published:** 2008

**Authors:** Indrapal Singh, Pankaj Verma, Kedar M. Agarwal

**Affiliations:** Department of Psychiatry, Safdarjang Hospital and V. M. Medical College, New Delhi, India. E-mail: dripsingh@rediffmail.com

Sir,

Mr. V a 26-year-old male was diagnosed as a case of resistant schizophrenia with obsessive compulsive disorder (OCD) and he showed significant improvement on clozapine 400 mgs after multiple trials of different antipsychotics. He started going to his job. His OCD was already well managed on flouxetine. However, Mr. V suffered from troublesome excessive salivation and sedation. The patient's parents were concerned with these side effects and requested to either stop or decrease the dose of medication. On the other hand, the patient was quite satisfied with his level of improvement. He was reluctant to stop the medication and face the risk of relapse. The clozapine was tapered to 300 mg/day and all the doses were shifted in the evening to manage side effects. Now there was no complaint of excessive salivation in day time. But the parents were still concerned about excessive salivation in the night and increased depth and duration of sleep (approximately 11-12 h). Amitryptaline and clonidine did not help much to reduce his salivation. He was advised to use towel under the pillow for excessive salivation in the night. According to the parents side effects are more pronounced than the level of improvement. However, the patient and resident in-charge noticed definite improvement in all the areas. The parents were educated again regarding the nature of his illness, level of improvement, role of clozapine and benign nature of side effects. However, even after repeated counselling, both mother and father kept on overemphasizing the side effects and insisting to either change or stop the clozapine. The parents revealed that these adverse effects may create problem if noticed by patient's wife after marriage, which was being planned in near future. It was planned to do detailed longitudinal evaluation of Mr. V in the presence his parents. On the basis of evaluation, the parents were made realized about the severity of the illness and impairment in the patient before the current treatment and then level of improvement in different areas (occupation, social interaction and self care) on standard scales after the use of clozapine. It was also explained that occasional expression of referential thinking by the patient is at the ideas level, not at the delusional level. The parents were told that patient's compliance is also a sign of improvement. The parents were undermining the benefits probably because the embarrassing side effects like excessive salivation which could be noticed by their prospective daughter-in-law. They were asked to anticipate the adverse consequences in patient's marital life including divorce if Mr. V relapsed on stopping clozapine.

At last this case could be managed successfully after repeated counselling and education of both the parents especially regarding their concern related to social issues like the patient's marriage.

Sir, in Indian context where the parent's decision in treatment is equally important, it is very important to convince and educate the key family members by different ways regarding the need of regular medications and about the side effects for successful management of schizophrenia.
